# Painless Ischemic Priapism Associated With Tamsulosin Use: A Case Report and Literature Review

**DOI:** 10.7759/cureus.111899

**Published:** 2026-07-01

**Authors:** Caitlin E Rector, Kevin Lei, Lael Hubbard, Navasard Ovasapians, Steven Sukin

**Affiliations:** 1 Pharmacy, Houston Methodist The Woodlands Hospital, The Woodlands, USA; 2 Emergency Medicine, Houston Methodist The Woodlands Hospital, The Woodlands, USA; 3 Internal Medicine, Houston Methodist The Woodlands Hospital, The Woodlands, USA; 4 Urology, Houston Methodist The Woodlands Hospital, The Woodlands, USA

**Keywords:** adverse drug events, alpha-blocker, clinical case report, ischemic priapism, tamsulosin, urological emergency

## Abstract

Tamsulosin, a selective α1-adrenergic antagonist prescribed for benign prostatic hyperplasia and expulsion of ureteral stones, carries a rare risk of priapism. We present a 59-year-old male with hypogonadism, hypertension, and hyperlipidemia who developed painless priapism 72 hours after initiating tamsulosin for ureterolithiasis. Despite the atypical absence of pain, penile blood gas analysis confirmed ischemic priapism. Initial treatment with intracavernosal phenylephrine (1000 mcg) failed, requiring bilateral corpus spongiosum shunts for resolution. A literature review revealed that many of the 14 existing cases identified occurred within 24 hours of drug initiation in middle-aged or older patients without additional risk factors. Approximately half responded to intracavernosal vasoconstrictors, while refractory cases required surgical intervention. To our knowledge, this is the first reported case of painless tamsulosin-induced ischemic priapism, emphasizing the importance of patient counseling and prompt evaluation of persistent erections regardless of pain intensity.

## Introduction

Tamsulosin is a selective α1-adrenergic antagonist widely prescribed to treat benign prostatic hyperplasia (BPH) and is used off-label for medical expulsive therapy of ureteral stones [1]. The drug has gained popularity due to its selectivity for α1A and α1D receptors in the prostate and ureter, which provides a therapeutic benefit with reduced cardiovascular side effects compared to non-selective α1-blockers [1-3].

Despite its favorable safety profile, tamsulosin carries the risk of priapism: a rare but serious adverse effect. According to product labeling, priapism associated with tamsulosin occurs at a frequency of 1 in 50,000 users, though it may be as high as 1 in 10,000 [4,5]. While this incidence is overall low, priapism represents a urological emergency that can result in permanent erectile dysfunction if not promptly treated [6,7]. Comparatively, the background incidence rate of priapism in the general population is estimated to be 0.5 to 0.9 cases per 100,000 person-years [5].

Ischemic priapism can be diagnosed with a cavernous blood gas and is characteristically associated with pain--a hallmark characteristic that differentiates it from non-ischemic priapism [7]. Ischemic priapism is considered a medical emergency and requires prompt intervention in order to attempt to preserve erectile function [6,7]. Misdiagnosis of ischemic priapism due to a lack of characteristic symptoms (namely pain) could lead to a delay in care and lasting erectile dysfunction.

We present a case of a patient who developed tamsulosin-induced painless ischemic priapism, confirmed by penile blood gas. To our knowledge, this is the first known report of a patient experiencing painless ischemic priapism, and one of the few reports of tamsulosin-induced priapism in the literature. We explore the unique presentation of painless ischemic priapism, discuss potential risk factors for the development of this adverse drug event, and summarize the available literature associated with this serious complication.

## Case presentation

A 59-year-old male with a past medical history of hypogonadism (on chronic injectable testosterone replacement therapy for 8 months), hypertension, and hyperlipidemia presented to the emergency department with a 4-hour persistent erection that began upon awakening. Three days prior, the patient had been diagnosed with ureterolithiasis after computed tomography (CT) revealed a 4 millimeter minimally obstructing stone at the right ureterovesical junction (Figure 1). Urinalysis at that visit was concerning for urinary tract infection. He was discharged on tamsulosin 0.4 mg daily, acetaminophen with codeine, ketorolac, ondansetron, and cephalexin.

**Figure 1:**
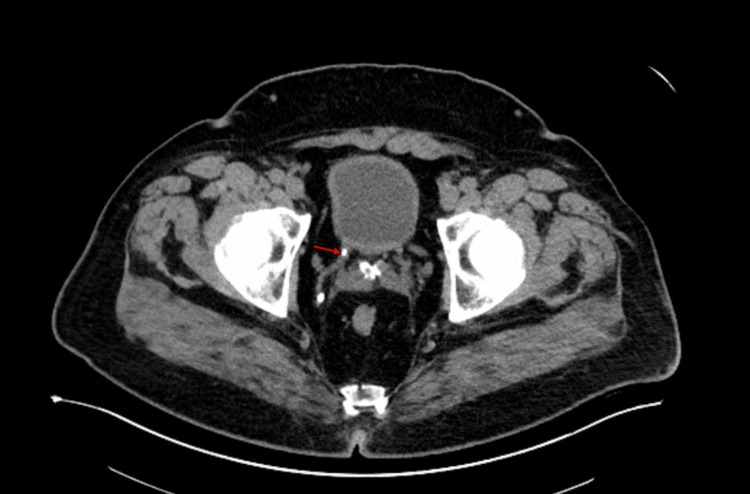
CT renal stone protocol CT demonstrates a 4 mm minimally obstructive stone (arrow) three days prior to the patient's admission for priapism.

The patient denied illicit drug use, penile trauma, recent sexual activity, urologic procedures, or penile injections. Physical examination was unremarkable, except for priapism. Notably, the priapism was non-painful in nature.

Laboratory studies, including a comprehensive metabolic panel, complete blood count, and coagulation studies, were within normal limits. Urinalysis showed trace ketones and microscopic hematuria. Urine drug screen was negative except for opioids, consistent with morphine administration in the emergency department. A cavernous blood gas analysis confirmed low-flow, ischemic priapism with severe acidosis (Table 1).

**Table 1:** Cavernous blood gas Results from the patient's cavernous blood gas collected in the emergency department prior to surgical intervention, including reference ranges for the diagnosis of ischemic priapism. pCO_2_: partial pressure of carbon dioxide; pO_2_: partial pressure of oxygen

Lab	Patient's result	Reference range for ischemic priapism [7,8]
pH	6.72	<7.25
pCO_2_ (mmHg)	156	>60
pO_2_ (mmHg)	3	<30

Initial management consisted of intracavernosal (IC) phenylephrine injection. The patient was placed on continuous cardiac monitoring, and a total of 1000 mcg phenylephrine (100 mcg/mL concentration) was administered bilaterally in escalating doses, consistent with guideline recommendations [6,7]: initial 200 mcg total (100 mcg per side), followed by 400 mcg, then an additional 400 mcg at 5-minute intervals. Unfortunately, the patient's erection persisted.

Given the unusual presentation of painless priapism coupled with the failure of phenylephrine to achieve detumescence, a CT angiogram of the abdomen and pelvis was performed to evaluate for vascular malformations, which could be contributing to the patient's symptoms. Imaging demonstrated multiple non-obstructing renal calculi with patent aortoiliac vasculature and no evidence of arteriovenous malformation (Figure 2).

**Figure 2:**
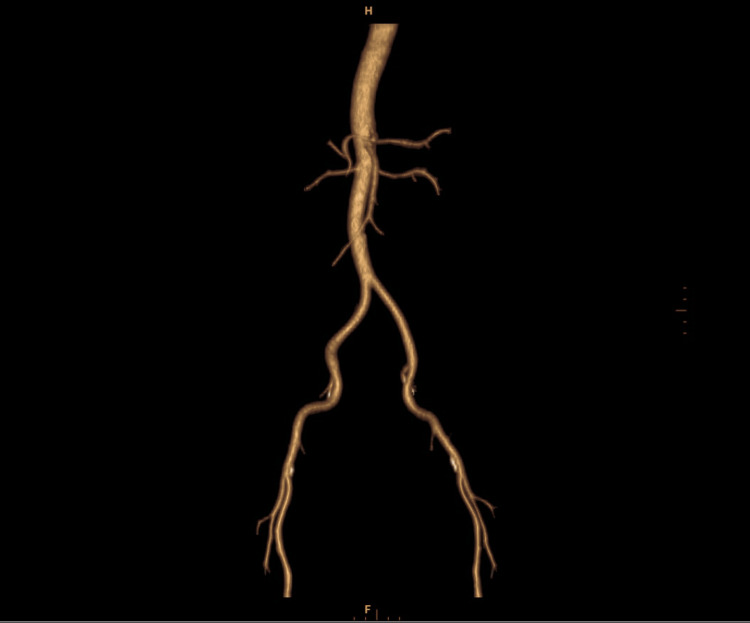
CT angiogram CT angiogram of the abdomen and pelvis demonstrates patent vasculature without arteriovenous malformation.

Despite maximal medical therapy, the priapism persisted without improvement. Further aspiration and irrigation were recommended; however, the patient declined this procedure in the emergency department, necessitating surgical intervention. While under general anesthesia, aspiration and irrigation were attempted, which did not resolve the priapism. Bilateral corpus spongiosum shunts were performed using the Winter procedure, successfully resolving the priapism. The patient's postoperative course was uncomplicated, and tamsulosin was discontinued.

At five months post-discharge, the patient reports continued difficulties obtaining and sustaining an erection. The patient provided written consent for the publication of this case report and expressed the hope that the publication of this case will help educate providers and prevent future patients from experiencing similar side effects from tamsulosin.

## Discussion

This case demonstrates a rare but clinically significant adverse effect of tamsulosin therapy. The proposed mechanism for α-blocker-induced priapism involves the inhibition of sympathetic effects necessary for penile detumescence through the blockade of α1-receptors, which are essential for normal erectile physiology [8,9]. While the exact mechanism remains unclear, priapism may arise due to pharmacological effects on the smooth muscle cells of the corpora cavernosa [8,10].

Our systematic review of reported cases (Table 2) reveals important patterns regarding the temporal relationship between tamsulosin initiation and priapism onset. Most strikingly, many of the cases occurred within 24 hours of starting tamsulosin or presented as recurrent erections after each dose [8,9,11-14]. This rapid or temporal onset suggests acute pharmacological sensitivity rather than cumulative drug effects. Our patient's presentation at 72 hours after starting tamsulosin, while slightly delayed compared to these reports, remains consistent with the established temporal pattern. A Naranjo Adverse Drug Reaction Probability Score of 7 indicates our patient experienced a probable adverse drug reaction to tamsulosin [15].

**Table 2:** Published cases of tamsulosin-induced priapism Upon review of the available literature, 14 unique cases of tamsulosin leading to priapism were identified. These cases are described here, including risk factors, onset of priapism, symptoms, treatment, and outcomes. BPH: benign prostatic hyperplasia, HCV: hepatitis C virus, HIV: human immunodeficiency virus, HLD: hyperlipidemia, HTN: hypertension, IC: intracavernosal, T2DM: type 2 diabetes mellitus *Interventions were not surgical in nature; however, they were performed in an operating room rather than the emergency department

First author	Year	Patient age (years)	Comorbidities or risk factors for priapism	Time from starting tamsulosin to onset of priapism	Duration of priapism at presentation	Reported symptoms	Cavernous blood gas	Treatments	Outcome
Mellado Castillero et al. [8]	2022	67	HTN, HLD, T2DM	One dose, less than 24 hours	Nine hours	Painful	pH 5.9	Surgical intervention*: Irrigation, IC phenylephrine	Full recovery
Mellado Castillero et al. [8]	2022	61	None	Three hours after first dose	48 hours	Painful	Described as confirming “low-flow priapism”	Non-surgical intervention: Irrigation, IC phenylephrine; Surgical intervention: Numerous shunt procedures without success	Erection gradually subsided over four days with supportive care; erectile dysfunction noted at follow-up
Spagnul et al. [9]	2011	32	Enlarged prostate	One dose, less than 24 h	40 hours	Painful	Not reported	Non-surgical intervention: Aspiration, irrigation, IC epinephrine	Full recovery
Kilinc et al. [10]	2009	59	Infravesical obstruction	Two hours after second dose	48 hours	Painful, only partial erection	Blood from thrombotic segment (pH 6.85, pCO2 84 mmHg, pO2 1.6 mmHg) and distally aspirated blood (pH 7.45, pCO2 29.8 mmHg, pO2 120 mmHg)	Surgical intervention: Irrigation, proximal corpus cavernosum-spongiosum shunt	Full recovery
Khater et al. [11]	2020	61	Paraplegia, HTN	One dose, less than 24 hours	18 hours	Painful	Not reported	Non-surgical intervention: Aspiration, irrigation, IC phenylephrine	Full recovery
Khater et al. [11]	2020	24	Ureteroscopy and laser lithotripsy 3 days prior	Less than 24 hours	72 hours	Painful	Not reported	Non-surgical intervention: Aspiration, irrigation, IC phenylephrine; Surgical intervention: Penoscrotal corporeal decompression	Reported loss of potency at follow-up
Pahuja et al. [12]	2005	56	BPH	Patient reported erections after each dose - had been on tamsulosin for two weeks prior to presentation	26-28 hours	Painful	Described as suggesting “low flow priapism”	Non-surgical intervention: Aspiration, irrigation, IC phenylephrine; Surgical intervention: Distal transglandular cavernosal shunt	Priapism persisted in the postoperative period
Prihadi et al. [13]	2020	57	HTN, HLD, benign prostatic obstruction	Not specified; patient experienced recurrent erections with tamsulosin daily use	72 hours	Painful	pH 6.87, pCO2 73 mmHg, pO2 1.4 mmHg, bicarbonate 13 mmol/L, SaO2 5%	Surgical intervention*: IC epinephrine, distal transglandular cavernosal irrigation and aspiration	Detumescence achieved
Cosentino et al. [14]	2015	67	BPH	Recurrent erections over two years of tamsulosin treatment – first episode three to four weeks after starting treatment	First episode: 16 hours Last episode: six hours	Painful	Last episode: pO2 9.7 mmHg, pCO2 89 mmHg	First episode: Non-surgical intervention: IC epinephrine, aspiration, irrigation; Surgical intervention: Winter shunt. Last episode: Non-surgical intervention: IC vasoconstrictors, irrigation	First episode: Detumescence, recurrent erections over next two; Last episode: Detumescence, discontinuation of tamsulosin, no further priapism episodes
Hammond et al. [16]	2014	44	HIV, HCV, HTN, BPH, hypogonadism, concurrent use of doxazosin, quetiapine, and boceprevir	Not stated; however, boceprevir was initiated nine days prior to priapism	72 hours	Painful	Not reported	Non-surgical intervention: Irrigation, IC phenylephrine; Surgical intervention: Distal shunt	Detumescence; decreased sexual function, which was improving at follow-up
Nishimura et al. [17]	2020	71	BPH	Five days	Not stated	Painful	pH 6.828, pCO2 132.8 mmHg	Non-surgical intervention: Aspiration, IC phenylephrine; Surgical intervention: Distal then proximal shunts	Detumescence, loss of erectile function
Dodds et al. [18]	2003	58	HTN	One hour after fourth dose	Seven hours	Painful	Not reported	Non-surgical intervention: Irrigation, IC phenylephrine	Detumescence – two weeks later patient resumed tamsulosin and experienced recurrent priapism, which was managed with the same treatment – after six months off alpha blockers, no recurrent priapism
Unal et al. [19]	2022	54	HTN, BPH	Four days	12 hours	Not reported	Not reported	Non-surgical intervention: Aspiration, IC epinephrine	Detumescence, no complications reported
Marconi et al. [20]	2019	45	Renal colic	After second dose	Six hours	Painful	Not reported	Non-surgical intervention: IC phenylephrine	Full recovery

Numerous risk factors exist for the development of ischemic priapism, including sickle cell disease, malignancy, pelvic or genital trauma, or use of certain drugs and medications [7]. In our review of the literature, the vast majority of patients did not have any additional risk factors apart from the use of tamsulosin; however, several shared common comorbidities including hypertension, diabetes mellitus, BPH, and hypogonadism [8,11-14,16-19]. Notably, advanced age appears to be a common factor, with most patients being middle-aged or older (median age 57.5 years, interquartile range 47.25 to 61). Our 59-year-old patient with hypogonadism, hypertension, and hyperlipidemia fits this demographic pattern, suggesting that age-related physiological changes may contribute to susceptibility.

Concomitant medications merit particular attention. One notable case involved drug interactions with boceprevir, a CYP3A4 inhibitor, and concomitant use of doxazosin. Boceprevir likely increased tamsulosin and doxazosin plasma concentrations and contributed to priapism development [16]. While our patient was not taking known interacting medications, the potential for drug interactions (namely, CYP3A4 and CYP2D6 inhibitors) should be considered when prescribing tamsulosin [4,16].

The duration of priapism in reported cases ranged from 6 to 72 hours, with our patient's 4-hour presentation representing a relatively brief episode [8-14,16-20]. Importantly, longer episodes were frequently associated with increased risk of erectile dysfunction or need for shunting procedures [8,11-14,16] as compared to cases in which patients presented within 10 hours of the onset of priapism [8,14,18,20]. Cases requiring surgical shunting procedures often resulted in compromised erectile function, emphasizing the importance of prompt recognition and early treatment [8,11,12,16,17].

Management approaches varied across cases but generally followed established protocols [6,7]. First-line treatment with IC vasoconstrictors (phenylephrine or epinephrine) was successful in approximately half of the cases [8-9,11,13,14,18-20], while refractory priapism required surgical intervention [8,10-12,14,16,17]. Our patient's requirement for surgical shunting after failed medical management aligns with current guideline recommendations, but it does demonstrate that despite early presentation after erection onset (four hours), surgical intervention may be necessary. This underlines the importance of counseling patients who are prescribed tamsulosin to seek prompt care for an erection lasting longer than four hours [7].

To our knowledge, this is the first case report of tamsulosin inducing painless priapism. Typically, ischemic priapism results in a painful erection, with pain being one of the hallmark characteristics differentiating it from non-ischemic priapism [6,7]. Our patient's case, as well as several cases in our review, support the fact that tamsulosin causes ischemic priapism, as demonstrated by cavernous blood gas analysis [8,10,12-14,17]. All cases of tamsulosin-induced priapism identified in our review reported painful erections apart from Unal et al. [19], who did not comment on symptomology (Table 2). While unclear why our patient did not report pain, this atypical presentation underscores the importance of maintaining clinical suspicion for priapism in patients recently initiated on tamsulosin, regardless of pain intensity.

This comprehensive review highlights the importance of patient counseling regarding priapism risk when prescribing tamsulosin, particularly in older patients with comorbidities or those taking concurrent medications that impact tamsulosin’s metabolism. The rapid onset observed in most cases suggests that patients should be educated to seek immediate medical attention for any persistent erection, especially within the first few days of initiating therapy.

## Conclusions

We report a case of painless ischemic priapism in a 59-year-old male who had been initiated on tamsulosin 72 hours prior to presentation. The temporal relationship between drug initiation and symptom onset, combined with the absence of other risk factors, strongly suggests causality. This case contributes to the limited literature on this rare but serious adverse effect and emphasizes the importance of patient education and prompt management when priapism occurs, even if the erection is painless. Clinicians should maintain awareness of this potential complication when prescribing α-blockers, particularly in patients with possible predisposing factors or drug interactions.

## References

[1] Nickel JC, Sander S, Moon TD (2008). A meta-analysis of the vascular-related safety profile and efficacy of alpha-adrenergic blockers for symptoms related to benign prostatic hyperplasia. Int J Clin Pract.

[2] Michel MC, Vrydag W (2006). α1-, α2- and β-adrenoceptors in the urinary bladder, urethra and prostate. Br J Pharmacol.

[3] Djavan B, Marberger M (1999). A meta-analysis on the efficacy and tolerability of alpha1-adrenoceptor antagonists in patients with lower urinary tract symptoms suggestive of benign prostatic obstruction. Eur Urol.

[4] (2025). Label: tamsulosin hydrochloride capsule. https://dailymed.nlm.nih.gov/dailymed/drugInfo.cfm?setid=d6e4cf51-e8cb-08c4-e053-2a95a90aefd7.

[5] Russom M, Fitsum Y, Debesai M, Russom N, Bahta M (2022). Tamsulosin and risk of priapism: a causality assessment using Austin Bradford Hill Criteria. Pharmacol Res Perspect.

[6] Salonia A, Eardley I, Giuliano F (2014). European Association of Urology guidelines on priapism. Eur Urol.

[7] Bivalacqua TJ, Allen BK, Brock G (2021). Acute ischemic priapism: an AUA/SMSNA guideline. J Urol.

[8] Mellado Castillero A, Gómez Gómez E, Campos Hernández JP, Prieto Castro R (2022). Physiopathology of the priapism secondary to tamsulosin: clinical cases of our hospital and literature review. Arch Esp Urol.

[9] Spagnul SJ, Cabral PH, Verndl DO, Glina S (2011). Adrenergic α-blockers: an infrequent and overlooked cause of priapism. Int J Impot Res.

[10] Kilinc M, Piskin M, Guven S, Gurbuz R, Odev K, Kaynar M (2009). Partial priapism secondary to tamsulosin: a case report and review of the literature. Andrologia.

[11] Khater U, Ramasamy R, Shah HN (2020). Tamsulosin-induced priapism: report of two cases and review of literature. J Endourol Case Rep.

[12] Pahuja A, Bashir J, Williamson EM, Barber N (2005). Unresolved priapism secondary to tamsulosin. Int J Impot Res.

[13] Prihadi JC, Kusumajaya C (2020). Priapism secondary to tamsulosin. A case report. Int J Surg Case Rep.

[14] Cosentino M, Sarquella J, Ruiz-Castane E (2015). Priapism secondary to tamsulosin: a case report. Rev Int Androl.

[15] Naranjo CA, Busto U, Sellers EM (1981). A method for estimating the probability of adverse drug reactions. Clin Pharmacol Ther.

[16] Hammond KP, Nielsen C, Linnebur SA, Langness JA, Ray G, Maroni P, Kiser JJ (2014). Priapism induced by boceprevir-CYP3A4 inhibition and α-adrenergic blockade: case report. Clin Infect Dis.

[17] Nishimura N, Torimoto K, Oda Y, Yoshida T, Uematsu T, Omori C, Fujimoto K (2020). A case report of severe ischemic priapism treated aggressively by proximal shunt [Article in Japanese]. Hinyokika Kiyo.

[18] Dodds PR, Batter SJ, Serels SR (2003). Priapism following ingestion of tamsulosin. J Urol.

[19] Unal S, Micoogullari U, Okulu E, Kayigil O (2022). Priapism - a rare side effect of alpha blockers: report of 2 cases and literature review. Rev Int Androl.

[20] Marconi M, Pavez P, San Francisco I, Narvaez P (2019). Priapism induced by use of tamsulosin: a case report and review of the literature. Arch Ital Urol Androl.

